# Cholecystectomy Is Linked to Worse Clinical Outcomes in Primary Sclerosing Cholangitis

**DOI:** 10.7759/cureus.80184

**Published:** 2025-03-06

**Authors:** Nozomi Miyake, Kengo Yasugi, Akinobu Takaki, Kazuyuki Matsumoto, Motoyuki Otsuka

**Affiliations:** 1 Department of Gastroenterology and Hepatology, Okayama University, Okayama, JPN; 2 Department of Gastroenterology and Hepatology, Okayama University Hospital, Okayama, JPN

**Keywords:** biliary diseases, cholecystectomy, gall bladder, liver function, post cholecystectomy, primary sclerosing cholangitis (psc)

## Abstract

Recent findings have suggested that gallbladder-derived retinoic acid signaling plays a crucial role in the regeneration of damaged intrahepatic biliary ducts. This retrospective cohort study analyzed the clinical records of 20 patients with primary sclerosing cholangitis (PSC) treated at our hospital between 2013 and 2024. We investigated the clinical implications of gallbladder removal in patients with PSC, a progressive cholangiopathy with limited therapeutic options. We retrospectively analyzed the data of patients with PSC and compared patients with and without prior cholecystectomy to assess the impact on disease progression using the Mayo risk score, Fibrosis-4 (FIB4) index, and other clinical parameters. Our findings indicated that cholecystectomy was associated with worse Mayo risk scores (p = 0.0004) and an elevated FIB4 index (p = 0.021), suggesting a potential link between gallbladder removal and accelerated disease progression. Furthermore, mortality and transplant-free survival analysis revealed significantly worse outcomes in the cholecystectomy group (odds ratio = 21.0, p = 0.032). However, given the retrospective nature and small sample size of this study, selection bias cannot be excluded, and further research is needed to confirm these findings. These findings support the hypothesis that gallbladder-derived factors, such as retinoic acid, may influence PSC progression and highlight the need for further research into therapeutic interventions targeting this pathway.

## Introduction

Primary sclerosing cholangitis (PSC) is a chronic progressive cholestatic liver disease characterized by inflammation, fibrosis, and stricturing of the intrahepatic and extrahepatic bile ducts [1-3]. It often leads to complications such as biliary cirrhosis, liver failure, and a higher risk of cholangiocarcinoma [4]. Despite ongoing research, effective medical treatments remain limited, and liver transplantation is the only definitive therapy for end-stage PSC.

Historically, the gallbladder has been regarded as a passive bile reservoir with little direct involvement in bile duct homeostasis or regeneration [5,6]. Conventional understanding suggests that bile flow regulation depends primarily on the liver and biliary epithelium, whereas the role of the gallbladder is limited to bile storage and controlled release [7]. Therefore, cholecystectomy has long been considered a procedure with minimal long-term impact on hepatobiliary function, aside from alterations in bile acid circulation. However, emerging evidence challenges this view, suggesting that the gallbladder actively contributes to biliary repair and disease modulation.

Bile duct repair mechanisms play a crucial role in PSC progression [8]. A recent study identified a previously unrecognized role of the gallbladder in bile duct regeneration: gallbladder-derived smooth muscle cells migrate to the common hepatic duct (CHD) following bile duct injury and produce retinoic acid (RA), which activates SOX9-mediated biliary epithelial cell proliferation and bile duct repair [9,10]. This finding raises the intriguing possibility that gallbladder removal (cholecystectomy) could impair biliary repair mechanisms, leading to accelerated disease progression in PSC [9,10].

To test this hypothesis, we conducted a retrospective analysis comparing patients with PSC with and without prior cholecystectomy. We assessed differences in Mayo risk scores, Fibrosis-4 (FIB4) index, and transplant-free survival to determine whether cholecystectomy was a risk factor for disease progression.

## Materials and methods

Study design, patient selection, and ethics

This retrospective cohort study was conducted using data from a hospital database containing clinical records of patients with PSC who were evaluated and treated at our hospital between 2013 and 2024. The exclusion criteria included patients who underwent liver transplantation before data collection, those with insufficient clinical records, and those lost to follow-up. The inclusion criterion was a confirmed diagnosis of PSC based on imaging, biochemical markers, and histological findings. Patients who underwent liver transplantation at the time of data collection were excluded. The remaining patients were stratified into two groups based on their cholecystectomy status. The cholecystectomy group comprised patients who underwent gallbladder removal prior to PSC diagnosis, whereas the non-cholecystectomy group comprised individuals who retained their gallbladder. However, the exact timing of cholecystectomy relative to PSC diagnosis was not available for all cases, which is acknowledged as a study limitation. This classification allowed for a comparative analysis of disease severity and progression between the two groups. Informed consent was obtained from all patients included in the study. The study protocol conformed to the ethical guidelines of the 1975 Declaration of Helsinki and was approved by the Ethics Committee of the Okayama University Hospital (Approval number: Ken1603-025). Patient consent was obtained via an opt-out system.

Clinical parameters

Several key clinical markers were analyzed and compared between the cholecystectomy and non-cholecystectomy groups to evaluate disease severity and progression. The Mayo risk score was calculated for each patient as it is a well-established prognostic model for PSC [11]. This score incorporated various clinical parameters, including age, bilirubin level, albumin concentration, aspartate aminotransferase (AST) level, and history of variceal bleeding, to predict the survival outcomes in patients with PSC.

In addition to the Mayo risk score, the FIB4 index was assessed as a measure of hepatic fibrosis [12]. The FIB4 index was calculated as previously described. A higher FIB4 index indicated more severe fibrosis, making it a valuable marker for assessing PSC-related liver damage.

Additional biochemical parameters, including AST, alanine aminotransferase (ALT), alkaline phosphatase (ALP), gamma-glutamyl transferase (gGTP), and platelet levels, were examined to provide a broader understanding of the disease state. Platelet count was monitored closely because of its inverse relationship with liver fibrosis, as lower levels suggest a more advanced disease status.

Statistical analyses

Statistical analyses were performed using GraphPad Prism 10 (GraphPad Software, San Diego, CA) to compare the clinical parameters between the cholecystectomy and non-cholecystectomy groups. Student's t-tests were used for normally distributed continuous variables, whereas the Mann-Whitney U test was applied to non-parametric data to determine statistical significance. The relationship between disease severity and hepatic fibrosis was further explored using Spearman correlation analysis to assess the association between the Mayo risk score and FIB4 index. A stronger correlation within a specific group suggested a more pronounced relationship between fibrosis progression and the overall disease severity. A 2 × 2 contingency table analysis was conducted to evaluate the effect of cholecystectomy on clinical outcomes. This analysis was used to compare the mortality and liver transplantation rates between the two groups, with odds ratios (ORs) calculated using Fisher's exact test to determine the relative risk of adverse outcomes.

## Results

Patient characteristics

Twenty patients diagnosed with PSC who visited our hospital were included (Table 1). Among them, five patients (5/20, 25%) had previously undergone cholecystectomy due to gallstone disease or cholecystitis, whereas 15 patients (15/20, 75%) retained their gallbladder. Although the median age differed between the two groups (46 vs. 63 years in the non-cholecystectomy and cholecystectomy groups, respectively), the difference was not statistically significant (p = 0.189). Similarly, the median follow-up period during which we observed the incidence of mortality and liver transplantation was longer in the non-cholecystectomy group (1,045 days) than in the cholecystectomy group (144 days), but this difference was not statistically significant (p = 0.073). Importantly, none of the patients underwent cholecystectomy during the observation period. In addition, albumin levels were significantly lower in the cholecystectomy group (median: 2.6 g/dl) than those in the non-cholecystectomy group (median: 4.4 g/dl, p = 0.005), suggesting a potential impact on liver function. Hemoglobin levels and platelet counts were comparable between the groups, with no significant differences observed (p = 0.204 and p = 0.895, respectively).

**Table 1:** Patient characteristics. Values are expressed as median (range). Values for categorical variables (male or non-male) are presented as n (%). *: Chi-square (χ²) value. Statistical values of all other parameters were determined using t-test. **: Statistically significant difference (p < 0.05). T. Bil: total bilirubin; PT-INR: prothrombin time-international normalized ratio; AST: aspartate aminotransferase; ALT: alanine aminotransferase; ALP: alkaline phosphatase.

	Cholecystectomy (-) (n = 15)	Cholecystectomy (+) (n = 5)	t-value	p
Follow-up period (day)	1045 (42 - 3304)	144 (34 - 1106)	3.72	0.073
Age, year	46 (25 - 76)	63 (43 - 74)	-3.56	0.189
Male (%)	8/15 (53)	4/5 (80)	0.28*	0.275
Hemoglobin (g/dl)	13 (11.4 - 15.7)	12.1 (8.9 - 16.2)	1.04	0.204
Platelet (× 104/μl)	27.2 (15.1 - 46.1)	26.5 (15.9 - 42.1)	0.20	0.895
Albumin (g/dl)	4.4 (3.7 - 4.8)	2.6 (2.2 - 4.2)	7.67	0.005**
AST (U/l)	48 (19 - 112)	84 (19 - 136)	-2.50	0.149
ALT (U/l)	56 (10 - 209)	106 (10 - 116)	-2.86	0.793
ALP (U/l)	493 (116 - 1057)	787 (343 - 1557)	-2.97	0.176
T. Bil (mg/dl)	0.71 (0.36 - 1.31)	5.2 (0.34 - 19.78)	-2.06	0.106
PT-INR	0.97 (0.83 - 1.07)	1.01 (0.90 - 3.29)	-0.15	0.353

Impact of cholecystectomy on liver fibrosis and liver function

A comparison of the clinical parameters between the cholecystectomy and non-cholecystectomy groups revealed a significant impact of cholecystectomy on liver fibrosis and liver function markers.

**Figure 1:**
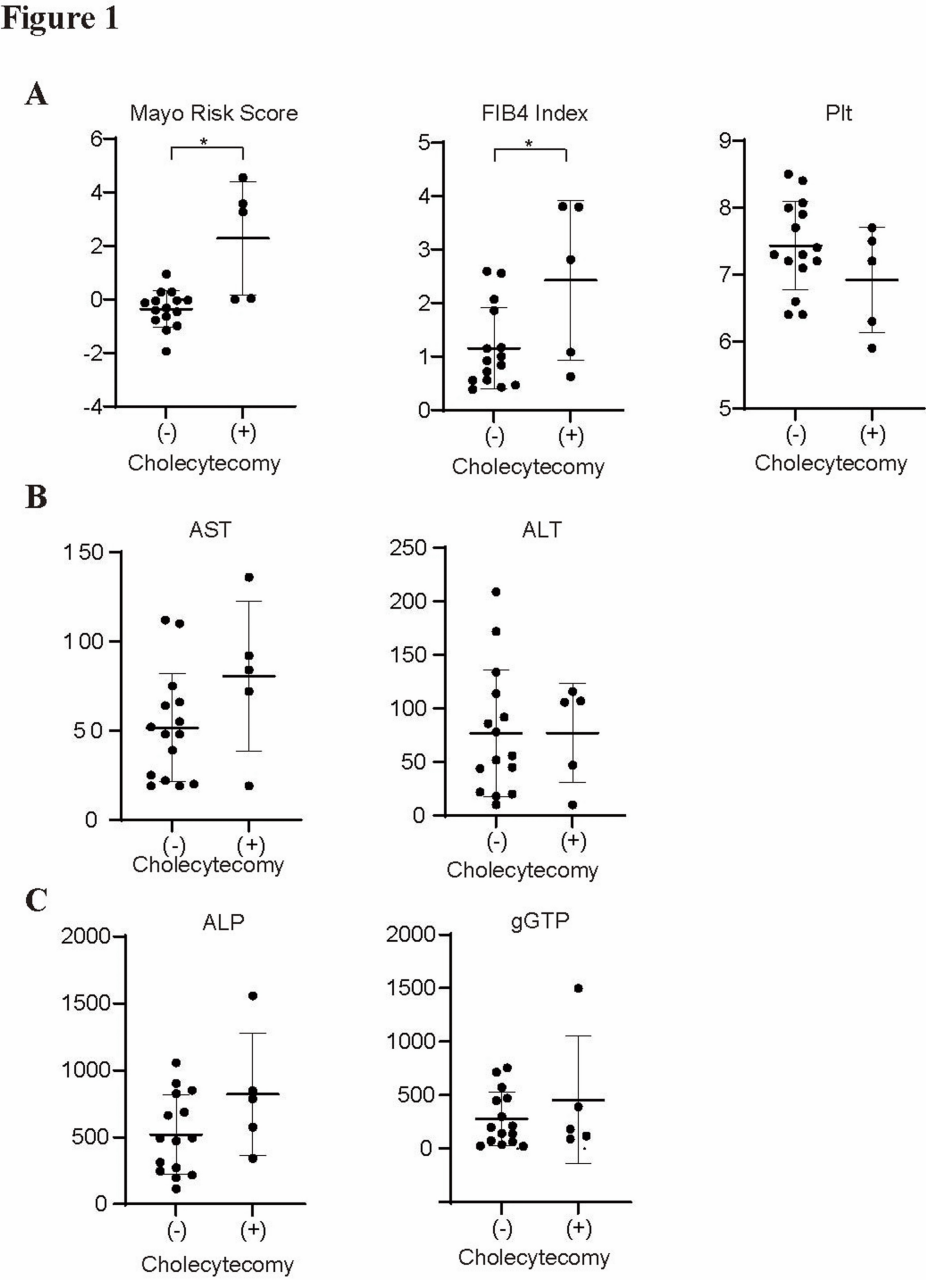
Comparison of clinical parameters between the cholecystectomy and non-cholecystectomy groups. (A) Dot plots with error bars comparing the Mayo risk score (left), Fibrosis-4 (FIB4) index (middle), and platelet count (Plt) (right) between patients with primary sclerosing cholangitis (PSC) (+) (n = 5) and without (-) (n = 15) prior cholecystectomy. Each dot represents an individual patient. Horizontal bars indicate mean values, and error bars represent standard deviations. Asterisks (*) indicate statistically significant differences (p < 0.05) between groups. (B) Dot plots illustrating aspartate aminotransferase (AST) (left) and alanine aminotransferase (ALT) (right) levels between the two groups. Each dot represents an individual patient. Horizontal bars indicate mean values, and error bars represent standard deviations. Asterisks (*) indicate statistically significant differences (p < 0.05) between groups. (C) Dot plots displaying alkaline phosphatase (ALP) (left) and gamma-glutamyl transferase (gGTP) (right) levels. Each dot represents an individual patient. Horizontal bars indicate mean values, and error bars represent standard deviations. Asterisks (*) indicate statistically significant differences (p < 0.05) between groups.

The Mayo risk score, a well-established prognostic indicator for PSC, was significantly higher in the cholecystectomy group (mean = 6.2 ± 1.8) than in the non-cholecystectomy group (mean = 4.7 ± 1.5, p = 0.0004) (Figure 1A), indicating a more severe disease state. Similarly, the FIB4 index, a surrogate marker for hepatic fibrosis, was markedly elevated in patients with prior cholecystectomy (mean = 5.3 ± 1.2) compared to that in the non-cholecystectomy group (mean = 3.6 ± 1.1, p = 0.021) (Figure 1A), suggesting a stronger fibrotic burden. Although platelet count, an indirect marker of portal hypertension and liver fibrosis, showed lower levels in the cholecystectomy group (mean = 110 × 10⁹/L) compared to those in the non-cholecystectomy group (mean = 155 × 10⁹/L), the difference was not statistically significant (p = 0.20) (Figure 1A). Additionally, liver function markers, including AST, ALT, ALP, and gGTP levels, exhibited mild differences between the two groups, with no statistically significant differences (Figures 1B, 1C). These findings suggest that prior cholecystectomy significantly affects liver fibrosis and function, whereas its effects on other clinical parameters are less pronounced.

Correlation between Mayo risk score and FIB4 index

**Figure 2:**
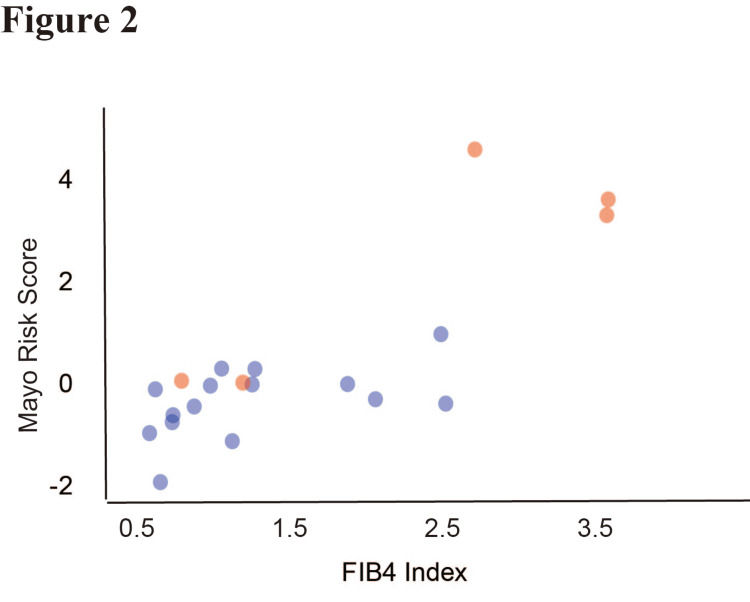
Correlation between the Mayo risk score and Fibrosis-4 (FIB4) index. Scatter plot showing the relationship between the Mayo risk score and FIB4 index. Patients with prior cholecystectomy (+) are marked in red (n = 5), whereas those without (-) prior cholecystectomy are marked in blue (n = 15). Spearman correlation analysis demonstrated a significant correlation between the Mayo risk score and FIB4 index (r = 0.65, p < 0.001). P < 0.05 was considered significant.

We performed a correlation analysis between the Mayo risk score and the FIB4 index to assess the relationship between liver fibrosis and overall disease severity. The Spearman correlation analysis demonstrated a strong positive correlation (r = 0.65, p < 0.001) (Figure 2); as PSC severity increased, the degree of hepatic fibrosis also increased.

Mortality and liver transplantation risk

The key objective of this study was to determine whether cholecystectomy is associated with poor long-term outcomes in patients with PSC. A 2 × 2 contingency table analysis revealed that among the five patients in the cholecystectomy group, two died (2/5, 40%) and one required liver transplantation (1/5, 20%) during the follow-up period. In contrast, in the non-cholecystectomy group, only one of the 15 patients (1/15, 6.7%) died, and no patients required liver transplantation. The odds ratio for mortality or transplantation in the cholecystectomy group was 21.0 (3/5 vs. 1/15, 60% vs. 6.7%, p = 0.032, Fisher's exact test) (Table 2), indicating a significantly elevated risk for adverse outcomes in these patients. The observation periods and causes of death in all cases are shown in Table 3.

**Table 2:** A contingency table for mortality and liver transplantation risk. The data have been represented as the number of cases. Death or transplantation (-): Patients who did not experience death or require transplantation. Death or transplantation (+): Patients who experienced death or required transplantation. Cholecystectomy (-): Patients who did not undergo cholecystectomy. Cholecystectomy (+): Patients who underwent cholecystectomy. Fisher’s exact test was used to assess the association between cholecystectomy and death or transplantation. A p-value < 0.05 was considered statistically significant.

	Death or transplantation (-)	Death or transplantation (+)
Cholecystectomy (+)	2	3
Cholecystectomy (-)	14	1

**Table 3:** Observation period and mortality causes for all cases. This table summarizes the observation period, survival status, and causes of death or liver transplantation for all patients included in the study. "Cholecystectomy" indicates whether the patient underwent gallbladder removal (+) or not (-). "Observation time (days)" refers to the duration from the patient's initial evaluation to the last follow-up or event. "Dead or Alive" represents the patient’s survival status at the end of the observation period. "Liver transplantation or cause of death" specifies whether the patient underwent liver transplantation or the cause of death in deceased cases. Cases without an entry in this column remained alive without requiring liver transplantation during the study period.

Case	Cholecystectomy	Observation time (days)	Dead or alive	Liver transplantation or cause of death
1	+	144	Dead	Liver failure
2	+	121	Alive	Transplantation
3	+	1,007	Dead	Biliary cancer
4	+	1,106	Alive	
5	+	34	Alive	
6	-	42	Alive	
7	-	420	Alive	
8	-	357	Alive	
9	-	2,518	Alive	
10	-	742	Alive	
11	-	449	Alive	
12	-	1,045	Alive	
13	-	1,078	Alive	
14	-	2,050	Dead	Biliary cancer
15	-	305	Alive	
16	-	1,165	Alive	
17	-	1,307	Alive	
18	-	1,018	Alive	
19	-	3,304	Alive	
20	-	2,044	Alive	

This substantial increase in risk suggests that gallbladder removal may contribute to disease progression beyond what is expected from the natural history of PSC.

## Discussion

Our study provides clinical evidence that prior cholecystectomy is associated with more severe PSC progression, as reflected by higher Mayo risk scores, higher FIB4 index, and worse transplant-free survival. However, given the retrospective design and the small number of cholecystectomy cases, definitive conclusions cannot be drawn, and the possibility of selection bias must be acknowledged. These findings are consistent with those of a recent study that revealed the key regenerative function of the gallbladder in bile duct repair [9]: gallbladder-derived smooth muscle cells migrate to the CHD following bile duct injury and produce RA, a signaling molecule critical for biliary epithelial cell proliferation [9,10]. Our data clinically suggest that the removal of the gallbladder in patients with PSC may deprive them of this regenerative mechanism, leading to a faster progression of fibrosis, worsened cholestasis, and an increased risk of mortality and transplantation.

Biliary repair failure is increasingly recognized as a driver of PSC progression [4,13]. A novel study showed that smooth muscle cell migration and intrahepatic bile duct regeneration failed in the absence of the gallbladder, resulting in persistent biliary injury. Considering PSC’s hallmark features of chronic inflammation, injury cycles, and progressive fibrosis [14], it is plausible that the loss of gallbladder-derived regenerative signaling accelerates disease progression. Our finding that patients who underwent cholecystectomy showed a stronger correlation between more severe progression and the Mayo risk score and FIB4 index supports the hypothesis that fibrosis progression is more tightly linked to overall disease severity in these patients, likely due to the absence of gallbladder-derived RA signaling.

A key finding of our study was the 21-fold increased risk of mortality or liver transplantation in patients with PSC who underwent cholecystectomy. This mirrors the experimental results [9,10] in which cholecystectomized mice exhibited impaired bile duct regeneration and increased susceptibility to biliary injury. Nonetheless, it remains uncertain whether cholecystectomy directly accelerates PSC progression or if patients requiring cholecystectomy inherently follow a more severe clinical course. Although some patients in our cohort may have undergone cholecystectomy before their PSC diagnosis because of gallstone disease, our results raise the question of whether gallbladder removal actively worsens PSC rather than being a consequence of early disease. Future prospective studies are needed to determine whether cholecystectomy is a modifiable risk factor for PSC.

Several mechanisms may explain why cholecystectomy worsens PSC outcomes. Retinoic acid is essential for bile duct differentiation and repair, and, as newly revealed, its absence following cholecystectomy may hinder regeneration, leading to unresolved biliary injury and progressive fibrosis. The gallbladder also plays a role in bile flow regulation; its removal alters bile acid metabolism and increases bile acid toxicity, thereby contributing to cholangiocyte apoptosis and hepatic fibrosis. In patients with PSC who are predisposed to biliary strictures and impaired bile flow, gallbladder removal may further exacerbate cholestatic liver injury. Additionally, bile acids influence the gut microbiota composition [15,16], and changes in bile acid circulation after cholecystectomy could contribute to dysbiosis, which has been linked to PSC pathogenesis [17]. Other gallbladder-derived regenerative factors may also be lost, warranting further investigation.

These findings suggest that cholecystectomy should be approached with caution in patients with PSC. Although gallbladder removal is often performed for gallstone disease, its potential impact on PSC progression may be more severe than previously recognized. Clinicians should carefully weigh the risks and benefits, particularly for early-stage PSC. For patients requiring cholecystectomy, alternative strategies should be considered to mitigate the loss of gallbladder-derived regenerative signaling. Potential therapeutic approaches include RA supplementation to restore lost signaling, bile acid-modulating agents such as nor-ursodeoxycholic acid to counteract altered bile acid homeostasis [18], and novel regenerative therapies targeting bile duct repair [19-21]. Future studies should determine whether specific interventions can mitigate the negative impact of cholecystectomy on PSC progression [22-24].

Although our study presented compelling evidence linking cholecystectomy to worse PSC outcomes, several limitations must be acknowledged. The retrospective design introduces a potential selection bias, and prospective validation is required. Additionally, our study lacked longitudinal biomarker data to assess whether changes in ALT, ALP, or total bilirubin levels were time-dependent following cholecystectomy. The exact timing of cholecystectomy relative to PSC onset remains uncertain, and although we included only cases in which cholecystectomy preceded PSC diagnosis, some patients may have undergone cholecystectomy due to early, undiagnosed PSC-related pathology. Additionally, RA was not directly measured in human bile ducts, preventing mechanistic confirmation. Finally, our sample size was relatively small. Future studies should focus on larger cohorts with prospective follow-ups to further investigate these associations. Larger multicenter studies are required to confirm these findings in different PSC subpopulations.

## Conclusions

In summary, our study provides the first clinical evidence that cholecystectomy is associated with worsened PSC progression, likely due to the loss of gallbladder-derived regenerative signals. These findings build upon recent experimental work, reinforcing the concept that the gallbladder plays a crucial role in bile duct homeostasis. Future studies should focus on identifying therapeutic strategies to compensate for the loss of gallbladder-derived factors in patients with PSC undergoing cholecystectomy, potentially paving the way for new targeted interventions.

## References

[1] Lazaridis KN, LaRusso NF (2016). Primary sclerosing cholangitis. N Engl J Med.

[2] Karlsen TH, Folseraas T, Thorburn D, Vesterhus M (2017). Primary sclerosing cholangitis - a comprehensive review. J Hepatol.

[3] Dyson JK, Beuers U, Jones DEJ, Lohse AW, Hudson M (2018). Primary sclerosing cholangitis. Lancet.

[4] Eaton JE, Talwalkar JA, Lazaridis KN, Gores GJ, Lindor KD (2013). Pathogenesis of primary sclerosing cholangitis and advances in diagnosis and management. Gastroenterology.

[5] Baron TH, Grimm IS, Swanstrom LL (2015). Interventional approaches to gallbladder disease. N Engl J Med.

[6] Housset C, Chrétien Y, Debray D, Chignard N (2016). Functions of the gallbladder. Compr Physiol.

[7] Lam R, Zakko A, Petrov JC, Kumar P, Duffy AJ, Muniraj T (2021). Gallbladder disorders: a comprehensive review. Dis Mon.

[8] De Assuncao TM, Jalan-Sakrikar N, Huebert RC (2017). Regenerative medicine and the biliary tree. Semin Liver Dis.

[9] He J, Li S, Yang Z (2025). Gallbladder-derived retinoic acid signalling drives reconstruction of the damaged intrahepatic biliary ducts. Nat Cell Biol.

[10] Wong W (2025). Rebuilding duct work from the gallbladder. Sci Signal.

[11] Kim WR, Therneau TM, Wiesner RH (2000). A revised natural history model for primary sclerosing cholangitis. Mayo Clin Proc.

[12] Vallet-Pichard A, Mallet V, Nalpas B (2007). FIB-4: an inexpensive and accurate marker of fibrosis in HCV infection. Comparison with liver biopsy and fibrotest. Hepatology.

[13] Tabibian JH, Masyuk AI, Masyuk TV, O'Hara SP, LaRusso NF (2013). Physiology of cholangiocytes. Compr Physiol.

[14] Reich M, Spomer L, Klindt C (2021). Downregulation of TGR5 (GPBAR1) in biliary epithelial cells contributes to the pathogenesis of sclerosing cholangitis. J Hepatol.

[15] Shin W, Kim HJ (2018). Intestinal barrier dysfunction orchestrates the onset of inflammatory host-microbiome cross-talk in a human gut inflammation-on-a-chip. Proc Natl Acad Sci U S A.

[16] Mousa OY, Juran BD, McCauley BM (2021). Bile acid profiles in primary sclerosing cholangitis and their ability to predict hepatic decompensation. Hepatology.

[17] Özdirik B, Schnabl B (2024). Microbial players in primary sclerosing cholangitis: current evidence and concepts. Cell Mol Gastroenterol Hepatol.

[18] Liu P, Jin M, Hu P (2024). Gut microbiota and bile acids: metabolic interactions and impacts on diabetic kidney disease. Curr Res Microb Sci.

[19] Assis DN, Abdelghany O, Cai SY (2017). Combination therapy of all-trans retinoic acid with ursodeoxycholic acid in patients with primary sclerosing cholangitis: a human pilot study. J Clin Gastroenterol.

[20] Floreani A, De Martin S (2021). Treatment of primary sclerosing cholangitis. Dig Liver Dis.

[21] Tan N, Lubel J, Kemp W, Roberts S, Majeed A (2023). Current therapeutics in primary sclerosing cholangitis. J Clin Transl Hepatol.

[22] Sohal A, Kowdley KV (2024). Novel preclinical developments of the primary sclerosing cholangitis treatment landscape. Expert Opin Investig Drugs.

[23] Maccauro V, Fianchi F, Gasbarrini A, Ponziani FR (2024). Gut microbiota in primary sclerosing cholangitis: from prognostic role to therapeutic implications. Dig Dis.

[24] Bhushan S, Sohal A, Kowdley KV (2025). Primary biliary cholangitis and primary sclerosing cholangitis therapy landscape. Am J Gastroenterol.

